# Metamaterial‐Enabled Hybrid Receive Coil for Enhanced Magnetic Resonance Imaging Capabilities

**DOI:** 10.1002/advs.202410907

**Published:** 2024-11-25

**Authors:** Xia Zhu, Ke Wu, Stephan W. Anderson, Xin Zhang

**Affiliations:** ^1^ Department of Mechanical Engineering Boston University Boston MA 02215 USA; ^2^ Photonics Center Boston University Boston MA 02215 USA; ^3^ Chobanian & Avedisian School of Medicine Boston University Medical Campus Boston MA 02118 USA

**Keywords:** metamaterials, magnetic resonance imaging, signal‐to‐noise ratio, radio frequency coils

## Abstract

Magnetic resonance imaging (MRI) relies on high‐performance receive coils to achieve optimal signal‐to‐noise ratio (SNR), but conventional designs are often bulky and complex. Recent advancements in metamaterial technology have led to the development of metamaterial‐inspired receive coils that enhance imaging capabilities and offer design flexibility. However, these configurations typically face challenges related to reduced adaptability and increased physical footprint. This study introduces a hybrid receive coil design that integrates an array of capacitively‐loaded ring resonators directly onto the same plane as the coil, preserving its 2D layout without increasing its size. Both the coil and metamaterial are individually non‐resonant at the targeted Larmor frequency, but their mutual coupling induces a resonance shift, achieving a frequency match and forming a hybrid structure with enhanced SNR. Experimental validation on a 3.0 T MRI platform shows that this design allows for adjustable trade‐offs between peak SNR and penetration depth, making it adaptable for various clinical imaging scenarios.

## Graphical abstract

This study presents a hybrid receive coil design that integrates metamaterials directly onto the coil's plane, maintaining a compact size while enhancing its imaging capabilities. By achieving a frequency match through mutual coupling, the coil achieves improved signal‐to‐noise ratio compared to a standalone coil. Experimental results on a 3.0 T MRI platform demonstrate its adaptability for different imaging scenarios.

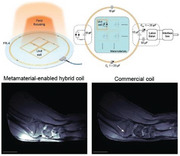

## 1 Introduction

Magnetic resonance imaging (MRI), a non‐invasive and non‐radioactive imaging modality, has emerged as a pivotal diagnostic tool in contemporary medicine, offering high‐resolution and precise visualization of tissue composition within the human body, facilitating rapid disease detection and effective treatment planning in clinical settings.^[^ ^1^, ^2^ ^]^ MRI relies on nuclear magnetic resonance, where nuclear spins undergo deliberate perturbation by an external excitation, leading to their subsequent relaxation while emitting the MRI signal.^[^ ^3^, ^4^ ^]^ This process critically relies on the radio frequency (RF) subsystem, comprising both transmit coils and receive coils, essential for generating and capturing the RF magnetic field required for MRI.^[^ ^5^ ^]^ Typically, a body coil (BC) embedded in the MRI bore serves as the transmit coil, producing a homogeneous excitation in the region of interest (ROI).^[^ ^6^, ^7^ ^]^ Meanwhile, dedicated receive coils or coil arrays, usually consisting of overlapping loops of conductive traces, are positioned proximal to the ROI to capture the MRI signal with minimal noise and provide substantial signal‐to‐noise ratio (SNR).^[^ ^8^, ^9^ ^]^

However, conventional receive coils are often cumbersome and heavy, as each coil element requires separate cabling, cable traps, feed boards, and pre‐amplifiers, escalating the overall construction cost, increasing setup time, and necessitating careful handling during routine imaging procedures.^[^ ^10^, ^11^, ^12^ ^]^ Recent endeavors to engineer flexible, conformable receive coils with alternative conductor trace options have sought to overcome these challenges.^[^ ^13^, ^14^, ^15^, ^16^, ^17^, ^18^ ^]^ Nevertheless, the performance of such conventional loop coils remains limited by their dimensions and RF layout.^[^ ^19^ ^]^

Innovations in wireless devices utilizing artificially constructed materials, such as metamaterials, have demonstrated viability for MRI applications. Specifically, these materials function in conjunction with the BC, which serves as both the transmit and receive coil, to amplify the MRI signal during reception, thereby increasing SNR.^[^ ^20^, ^21^, ^22^, ^23^ ^]^ While this approach eliminates the need for heavy and bulky receive coil arrays, it suffers from reduced sensitivity and SNR due to the predominant physical separation between the ROI and the BC's conducting traces. Ongoing efforts aim to optimize the metamaterial construction strategies to enhance performance, incorporating increasingly advanced features like anatomic‐specific designs,^[^ ^24^, ^25^, ^26^ ^]^ flexible and conformable configurations,^[^ ^27^, ^28^, ^29^ ^]^ and minimized dielectric losses.^[^ ^30^, ^31^, ^32^, ^33^ ^]^ Consequently, the latest advancements in metamaterial technology for MRI have shown promise in achieving SNR levels comparable to or surpassing those of the state‐of‐the‐art receive coil arrays, making them increasingly viable for clinical use.^[^ ^32^, ^33^ ^]^

Naturally, leveraging the metamaterials’ field enhancement capability in the near‐field region also presents a promising opportunity to enhance the imaging performance of existing receive coils. Recently, a series of metamaterial‐inspired receive coils have emerged,^[^ ^34^, ^35^, ^36^, ^37^, ^38^ ^]^ featuring metamaterial devices tailored to match the Larmor frequency of MRI platforms ranging from 7.0 T to 17.2 T, coupled with non‐resonant receive coils for signal acquisition, introducing additional flexibility in coil design. This integration has facilitated improvements in transmitting field homogeneity,^[^ ^34^ ^]^ enabled dual‐nuclei MRI,^[^ ^35^, ^36^ ^]^ and expanded the field of view (FOV) for covering regions beyond the reach of conventional loop coils.^[^ ^37^, ^38^ ^]^ However, these strategies often result in highly case‐specific configurations that may not be adaptable to existing receive coils. Additionally, efforts to combine resonant receive coils with non‐resonant metamaterials functioning as capacitive impedance surfaces have shown local SNR enhancement but require a predetermined physical arrangement between the coil, metamaterial, and imaging subject, limiting efficiency in practice.^[^ ^39^, ^40^ ^]^ Moreover, these strategies for integrating metamaterials with receive coils often transform a 2D coil configuration into a bulkier 3D setup, reducing compatibility with diverse imaging subjects. Thus, an effective strategy for seamlessly incorporating metamaterial technology into existing receive coils remains elusive.

In this paper, we introduce an approach for designing a metamaterial‐enabled hybrid receive coil by directly patterning the metamaterial, consisting of an array of capacitively‐loaded ring resonators, on the same plane as the existing coil within its coverage area. This method preserves the coil's 2D layout without increasing its physical footprint or compromising its original compatibility. In the proposed hybrid coil, both the metamaterial and the coil are individually non‐resonant at the targeted Larmor frequency. However, by incorporating the metamaterial into the coil's RF layout, they mutually couple to induce a resonance frequency shift, creating a hybrid structure that resonates at the Larmor frequency. This integration effectively redistributes and amplifies the magnetic field within the coil volume, achieving a substantially higher SNR level than conventional loop coils of the same coverage. Furthermore, we experimentally demonstrate on a 3.0 T MRI platform that the hybrid coil can be reconfigured by adjusting the mutual coupling between the metamaterial and the coil. This flexibility allows for optimization of the trade‐off between peak SNR and penetration depth, enabling optimization for various imaging scenarios.

## 2 Results

### 2.1 Hybrid Coil Design

**Figure 1:**
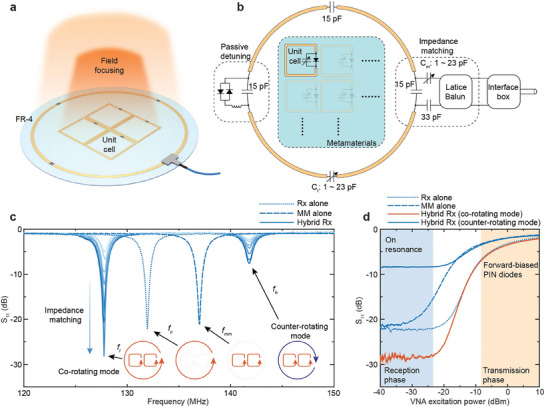
Concept of the metamaterial (MM)‐enabled hybrid receive coil (Rx). a) The hybrid coil is designed by patterning the metamaterial on the same plane and within the coverage of the coil loop, resulting in a field‐focusing effect within the coil's imaging volume, improving the SNR. b) Schematic of the hybrid coil layout. The coil circuit includes three distributed capacitors, one tuning capacitor, a passive detuning circuit, and a matching circuit. Each metamaterial unit cell contains a PIN diode and a trimmer capacitor. c) Measured reflection coefficient (*S_11_*) indicating the resonance frequencies of the coil alone (*f_c_*), the metamaterial alone (*f_mm_*), and the co‐rotating mode (*f_l_*) and counter‐rotating mode (*f_h_*) in the hybrid coil. Insets: current directions in the conductors for the associate resonance modes. d) Measured reflection coefficient at the resonance frequencies of the associate modes as a function of the excitation power from the VNA.

The imaging capabilities of the metamaterial‐enabled hybrid receive coil are primarily characterized by the SNR, expressed as follows:^[^ ^41^, ^42^ ^]^

(1) $$SNR\propto \frac{\omega^{2}B_{c}}{\sqrt{R_{coil}+R_{sample}}}$$

where ω is the Larmor frequency for MRI, and *B_c_* is the magnetic field produced by the current in the receive coil. The resistance of the receive coil is denoted by *R_coil_*, while the equivalent resistance of the sample in the coil is represented by *R_sample_*. The magnetic field term *B_c_*, often referred to as the coil sensitivity, is strongly correlated with the coil trace layout and is inversely proportional to the coil's size.^[^ ^19^, ^43^ ^]^ A key attribute of electromagnetic metamaterials is their ability to enhance the near‐field magnetic field due to the small dimensions of their unit cells. The assembly of multiple unit cells enables the metamaterial to achieve both strong field enhancement and expanded coverage, improving penetration depth through the superimposition of magnetic fields across the unit cells—a result of their collective working modes. By incorporating metamaterials with unit cell dimensions smaller than the coil itself, a field‐focusing effect can be achieved within the hybrid coil's sensitivity map, resulting in a locally enhanced SNR (**Figure** **1**a). This configuration is particularly advantageous for close‐to‐surface regions, including musculoskeletal imaging applications in wrist, elbow, ankle, and knee, where the required penetration depth is only a few centimeters.

To implement this coil design, a circular single‐channel loop coil with a diameter of 150 mm is constructed on an FR‐4 substrate, with the coil trace layout depicted in Figure 1b. The coil conductor trace is segmented by distributed capacitors (Knowles Syfer) for frequency matching to 127.7 MHz, the Larmor frequency for the 3T MRI platform used in this study. Additionally, a trimmer capacitor (1–23 pF, NMAM25HV, Knowles Voltronics) allows for a resonance frequency tuning range between 100 to 200 MHz, ensuring frequency alignment both with and without metamaterial integration. A pi matching network containing a lattice balun is included to achieve optimal impedance matching.^[^ ^44^, ^45^ ^]^ For passive detuning during the MRI transmission phase, a pair of PIN diodes (MADP‐000235‐10720T, MACOM Technology Solutions) along with a series inductor (JLC05E088TRSM, Knowles Johanson Manufacturing) are placed in parallel with a distributed capacitor.

The metamaterial is pattern on the same printed circuit board as the coil and is positioned at the center of the loop coil. Each unit cell of the metamaterial is integrated with a trimmer capacitor (1–23 pF), enabling a frequency tuning range between 100 and 300 MHz, and includes a PIN diode for passive detuning. This detuning mechanism remains activated if the current in the receive coil is insufficient to forward bias the diode pair within the coil circuit. For the ease of understanding the resonance mode behavior in the hybrid structure, we incorporate a 1 × 2 metamaterial array, which is sufficient for generating a collective resonance mode, in which the oscillating currents in all unit cells are in phase, leading to constructive interference and a significant magnetic field enhancement due to the synergy of individual unit cells. Upon assembling the metamaterial and the coil, the effect of electromagnetic coupling between them must be considered, as this coupling strongly deviates the resonance frequency of both the metamaterial alone (*f_mm_*) and the coil alone (*f_c_*) according to the coupled resonator theory.^[^ ^46^, ^47^ ^]^ Consequently, both the metamaterial and the coil must be individually tuned off‐resonance to ensure that the coupling‐induced mode splitting gives rise to an on‐resonance operating mode for the hybrid coil structure. The metamaterial and coil are arranged in a co‐planar configuration, with substantial overlap of their magnetic flux when on resonance. As a result, their mutual coupling is expected to be predominantly inductive. To elucidate the resonance modes of the ultimate hybrid coil, electromagnetic theory is applied to define the effective inductance *L* for either the metamaterial or the coil as follows:^[^ ^48^ ^]^

(2) $$L_{ij}=\frac{\mu_{0}}{4\pi |I_{i}I_{j}|}\int \;\;\;\int dr_{i}r_{j}\frac{J\left(r_{i}\right)\cdot J\left(r_{j}\right)}{|r_{i}-r_{j}|}$$

where *r_i_* and *r_j_* denote the integration elements along the conductor traces, *J*(*r_i_*) and *J*(*r_j_*) are the spatial current densities at *r_i_* and *r_j_*, and *I_i_* and *I_j_* are the equivalent electric currents in the respective objects. Equation (2) represents the self‐inductance of the metamaterial or the coil when *i* and *j* are equal, and mutual inductance between the metamaterial and the coil when *i* and *j* differ. The current distribution within each metamaterial unit cell follows a sinusoidal profile, while the current in the coil is approximated as uniform due to its multi‐segment conductor traces.^[^ ^49^ ^]^ The coupling coefficient between the coil and metamaterial is then expressed by:^[^ ^50^ ^]^

(3) $$k=\frac{L_{mutual}}{\sqrt{L_{m}\times L_{c}}}$$

where *L_m_* and *L_c_* represent the self‐inductance of the metamaterial and coil, respectively, while *L_mutual_* denotes their mutual inductance. In the adopted configuration for the hybrid coil, where the metamaterial and coil are both co‐planar and coaxial, the coupling coefficient is calculated to be consistently positive. This positive value aligns with observations in similar near‐field wireless power transfer systems where two inductors are arranged coaxially.^[^ ^47^, ^48^ ^]^ This mutual coupling explains the mode splitting observed in the hybrid structure, resulting in a shifted resonance frequency for a lower mode (*f_l_*) that is lower than either *f_mm_* or *f_c_*, and a higher mode (*f_h_*) that is higher than either *f_mm_* or *f_c_* (Figure 1c). According to the dynamics of coupled resonators,^[^ ^47^ ^]^ the positive coupling coefficient results in distinct behaviors for the lower and upper modes. For the lower mode, the currents in the metamaterial and coil are always in phase, referred to as the co‐rotating mode. This mode is desirable for the hybrid coil as it allows for the constructive superposition of the inductive magnetic fields from both the metamaterial and coil. Conversely, for the upper mode, referred to as the counter‐rotating mode, the currents in the metamaterial and coil are 180° out of phase, leading to destructive interference and creating a spatial phase transition region. This phase transition can result in a zero SNR gain, as will be discussed further.

By leveraging the mutual coupling between the metamaterial and the coil, it is possible to tune both *f_mm_* and *f_c_* to frequencies >127.7 MHz, thereby aligning the frequency of the desired co‐rotating mode *f_l_* with 127.7 MHz through mode splitting. Importantly, since the metamaterial is patterned and integrated into the coil layout and electromagnetically coupled with the coil, it behaves simply as mutual inductance in the coil circuit. Consequently, the impedance matching circuit of the coil remains effective in the hybrid configuration, as demonstrated by the reflection coefficient ($S_{11}$) measurements obtained using a vector network analyzer (VNA, P5020B Keysight Inc.) (Figure 1c). The $S_{11}$ of the co‐rotating mode can be optimized to below −25 dB, ensuring impedance match even after the metamaterial integration. Furthermore, the $S_{11}$ of the coil alone, the metamaterial alone, and the two modes in the hybrid coil are measured as a function of the VNA excitation power at their respective resonance frequencies (Figure 1d). As the excitation power gradually increases, which may reflect the typical high‐power RF transmitting fields of kilowatt‐level,^[^ ^51^ ^]^ all components‐the coil alone, the metamaterial alone, and the hybrid coil‐exhibit a tendency to cease resonating. As both the coil and metamaterial are equipped with passive detuning mechanisms, this ensures that the hybrid coil remains adaptable to the alternating field strengths during MRI image acquisition, facilitating its seamless integration with existing MRI platforms.

### 2.2 Electromagnetic Characterization

**Figure 2:**
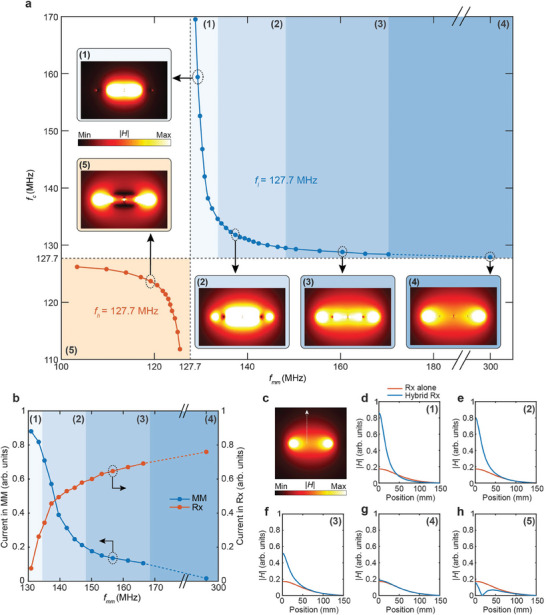
Electromagnetic characterizations of the hybrid coil. a) Combinations of the measured individual resonance frequencies of the metamaterial (*f_mm_*) and the coil (*f_c_*) that result in at least one resonance mode at 127.7 MHz for the hybrid coil. Inset: simulated side view of the magnetic field (|*H*|) distributions of the hybrid coil at 127.7 MHz for different combinations of *f_mm_* and *f_c_*. b) Simulated current amplitudes in the hybrid coil, on the metamaterial conductor and on the loop coil conductor, respectively. The hybrid coil is tuned and matched to 127.7 MHz for all configurations. c) Simulated magnetic field distribution of the coil alone at 127.7 MHz. d–h) Comparison of the hybrid coil's magnetic field profiles under five different cases with the coil alone, along the white dashed arrow in (c).

Matching the hybrid coil's resonance modes to the Larmor frequency does not require a specific combination of *f_mm_* and *f_c_*. Instead, within the tuning range of *f_mm_* (100–300 MHz), the receive coil's tuning and matching circuit can always be adjusted to yield an optimal *f_c_*, ensuring that the hybrid coil's working mode aligns with the Larmor frequency. **Figure** **2**a shows the measured combinations of *f_mm_* and *f_c_* that result in at least one resonance mode of the hybrid coil (either *f_l_* or *f_h_*) being tuned to 127.7 MHz. The relationship between *f_mm_* and *f_c_* resembles the pattern of a reciprocal function: when either *f_mm_* or *f_c_* approaches 127.7 MHz, the other component must be adjusted further away from 127.7 MHz. This adjustment reduces the extent of mode splitting required to maintain the frequency alignment of the hybrid structure. To tune the co‐rotating mode of the hybrid coil (*f_l_*) to the working frequency, both *f_mm_* and *f_c_* are set to be higher than 127.7 MHz. This setup ensures that the currents in the metamaterial and the coil interfere constructively, generating a focused magnetic field in the central volume of the coil. The magnetic field profiles of the hybrid coil, based on the resonance status of the metamaterial and coil alone, can be categorized into different cases that may offer distinct benefits for MRI applications.

When *f_mm_* is substantially closer to 127.7 MHz than *f_c_* (case (1)), the metamaterial dominates in the hybrid coil, while the coil, resonating at a much further frequency, has less impact. This configuration results in a strongly concentrated magnetic field in proximity to the metamaterial, with minimal contribution from the coil due to the significant difference in the currents between the two components at this frequency range (Figure 2b). Compared to the magnetic field generated by the coil alone (Figure 2c), this setup achieves up to a fivefold field enhancement at the coil surface, with this enhancement penetrating 45 mm along the coil's normal direction (Figure 2d).

When *f_mm_* and *f_c_* are more comparable (case (2)), a balance between the metamaterial's and the coil's contributions is achieved. In this intermediate scenario, the currents in the metamaterial and coil are more comparable (Figure 2b), allowing the hybrid coil to leverage the strong, focused magnetic field enhancement provided by the metamaterial, due to its smaller size, as well as the extended FOV and improved penetration depth of the coil, due to its larger dimension. The hybrid coil shows up to a 4.6‐fold field enhancement at the surface, with this enhancement penetrating 90 mm along the coil's normal direction (Figure 2e).

As *f_mm_* increases further (case (3)), the desired *f_c_* value approaches 127.7 MHz, causing the coil to become dominant while the metamaterial's influence diminishes. As the hybrid coil transitions into this region, the current in the coil increases steadily, whereas the current in the metamaterial decreases. Consequently, the magnetic field profile of the hybrid coil starts to resemble that of the coil alone, with the coil exhibiting a field enhancement of threefold at the surface and penetrating 120 mm along the coil's normal direction (Figure 2f). In this configuration, the hybrid coil improves its penetration depth at the expense of the peak field enhancement compared to case (2).

In case (4), with *f_mm_* adjusted to its upper limit of 300 MHz, under which its impact on the coil is minimal, resulting in a desired *f_c_* of 127.9 MHz. In this configuration, the mutual coupling induces only a slight resonance frequency shift of 0.2 MHz in the hybrid structure. The current in the metamaterial is negligible, and it exerts little effect on the coil's magnetic field profile (Figure 2g). This configuration demonstrates that, despite the potential for enhanced imaging capabilities through metamaterial integration, the hybrid coil can always be reconfigured to minimize the metamaterial's influence, thereby entirely preserving the coil's original imaging performance.

Finally, the frequency range where both *f_mm_* and *f_c_* are <127.7 MHz (case (5)) is also included, resulting in the counter‐rotating mode (*f_h_*) being tuned to the Larmor frequency. As anticipated, the out‐of‐phase currents in the metamaterial and coil interfere destructively, creating a phase transition region marked by the dark bands in the magnetic field profile. The magnetic field drops to zero in these regions (Figure 2h), rendering this field pattern unsuitable for MRI imaging operations.

## 3 MRI Validation

### 3.1 Phantom Validation

**Figure 3:**
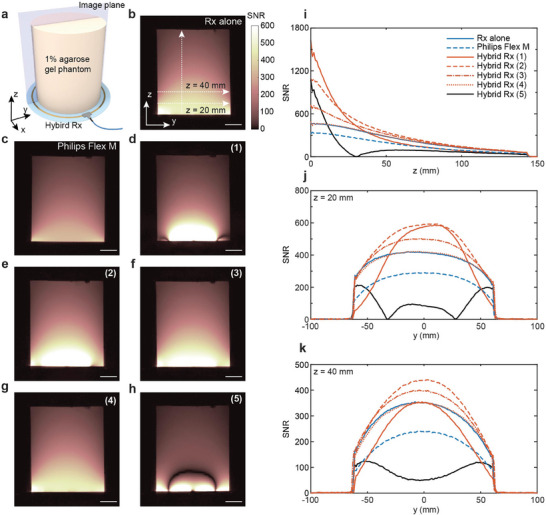
MRI validations with phantom. a) Experimental setup for phantom imaging using the hybrid coil. b–h) SNR maps acquired using the coil alone (b), the Philips dStream Flex M coil (c), and the hybrid coil under cases (1) to (5) (d–h). i–k) Comparison of SNR profiles along the z direction (i), and along the y direction with distances of 20 mm (j) and 40 mm (k) from the phantom surface. Scale bars in (b–h) are 3 cm.

To evaluate the performance of the hybrid coil in MRI, phantom imaging was conducted using the experimental setup shown in **Figure** **3**a. The phantom contains 1% agarose gel in a cylindrical mold and was placed directly on top of the coil circuit board with no physical separation to obtain the transverse MRI image. The hybrid coil was optimized for all five cases depicted in Figure 2a by adjusting the tuning and matching circuits in the coil, as well as the tuning capacitors in each metamaterial unit cell. To put the performance of the hybrid coil into perspective, SNR maps for each case were compared with those acquired using an identical coil without metamaterial integration and a commercial single‐channel receive coil (Philips dStream Flex M) with the same coverage (150 mm) as the hybrid coil (Figure 3b–h). The SNR profiles were compared along the coil's normal direction at the center of one metamaterial unit cell, and laterally across the phantom at distances of 20 and 40 mm away from the coil plane, respectively (Figure 3i–k).

For case (1), where the metamaterial takes dominance in the hybrid coil and substantially focus the magnetic field in its vicinity, an SNR enhancement of 3.54‐fold and 5.05‐fold is observed at the phantom surface compared to the coil alone and the commercial coil, respectively. This enhancement gradually decreases, extending up to 39 and 85 mm into the phantom, respectively (Figure 3i). Along the y‐axis, it is evident that this substantial SNR enhancement at the phantom surface comes at the expense of reduced coverage (Figure 3d). The current in the coil is relatively low, producing an insufficient magnetic field outside the metamaterial region, resulting in a focused SNR profile only in the area covered by the metamaterial (Figure 3j,k). Nevertheless, this configuration is advantageous for imaging volumes close to the surface where only a few centimeters of penetration depth are required. The metamaterial's field focusing effect provides higher SNR levels beyond the capability of the conventional receive coil alone.

Case (2) offers the optimal compromise between the improved penetration depth and high SNR at shallow depth, leveraging both the high SNR enhancement from the metamaterial and the extended coverage from the coil. The hybrid coil exhibits a lower SNR enhancement of 2.35‐fold and 3.37‐fold compared to the coil alone and the commercial coil, respectively. However, this enhancement persists throughout the entire 140 mm‐high phantom (Figure 3i). Additionally, this configuration does not compromise the coverage along the y‐axis, as evidenced in Figure 3j,k, where the SNR of the hybrid coil in Case (2) remains higher than that of the coil alone throughout the phantom. In contrast to case (1), this setup enables the hybrid coil to achieve an improved SNR not only at the phantom surface but also in regions up to 140 mm deep and beyond, achieving an improvement of sensitive volume while maintaining efficient SNR over the surface regions.

For case (3), where the coil is dominant in the hybrid coil, the peak SNR enhancement further decreases to only 1.57‐fold and 2.24‐fold compared to the coil alone and commercial coil, respectively. This enhancement also extends throughout the phantom along the normal direction, while outperforming case (2) beyond 130 mm into the phantom (Figure 3i). Similarly, the coverage along the y‐axis remains unaffected, as the coil, which carries a stronger current in this configuration, is the primary contributor to the coverage. Despite the trade‐off between the peak SNR enhancement and the penetration depth being evident when comparing case (2) and (3), the higher SNR observed at depths of 130 mm and beyond provides minimal practical value from the diagnostic perspective. Consequently, case (3) is considered suboptimal compared to case (2).

In case (4), where the metamaterial's resonance *f_mm_* is substantially detuned from 127.7 MHz, its effect on the hybrid coil's SNR map is negligible. The SNR profiles in Figure 3i–k coincide well with those of the coil alone. Since the metamaterials are patterned on the same circuit board as part of the coil's conductor, without requiring additional space, the hybrid coil effectively functions as if the metamaterials are absent, preserving the coil's original imaging capability.

Lastly, in case (5), the counter‐rotating mode is tuned to 127.7 MHz. Despite a concentrated SNR is also observed near the metamaterial, the SNR rapidly reduced to zero in the phase transition region, as anticipated, rendering this configuration unsuitable for any imaging applications. It should be noted that all the phantom experiment results have yielded SNR maps that align with the simulated magnetic field distribution for all five cases, validating the strong correlation between the hybrid coil's sensitivity map and the ultimate SNR gain.

### 3.2 Ex Vivo Validation

**Figure 4:**
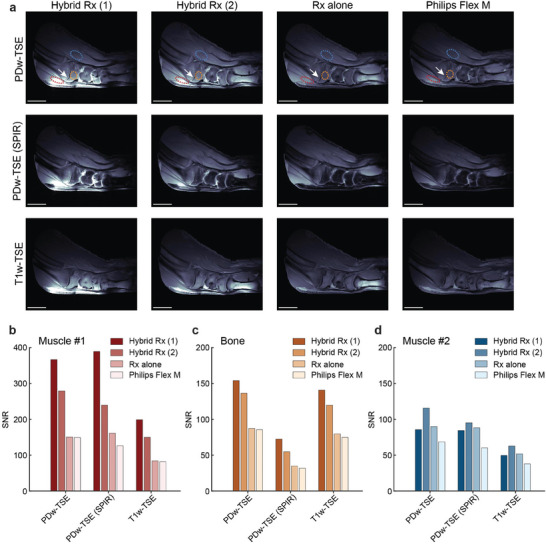
MRI validations with an ex vivo porcine leg. a) MRI images of the porcine leg acquired with the hybrid coil, the coil alone, and the Philips dStream Flex M coil using three pulse sequences: PDw‐TSE, PDw‐TSE (SPIR), and T1w‐TSE, normalized to the same noise level for each sequence. b–d) Quantitative evaluation of the SNR for different regions as outlined in (a). Scale bars in (a) are 3 cm.

To better visualize the hybrid coil's performance in a more biomedically relevant setting, experiments were conducted using an ex vivo porcine leg. Three pulse sequences were employed: proton density‐weighted turbo spin echo (PDw‐TSE), proton density‐weighted turbo spin echo with fat saturation (PDw‐TSE (SPIR)), and T1‐weighted turbo spin echo (T1w‐TSE). The hybrid coil incorporates a passive detuning feature in both the coil loop and each metamaterial unit cell, ensuring it remains consistently detuned regardless of the coil configuration or the selected sequences. The absence of artifacts in the resulting MRI images confirms the hybrid coil's compatibility with these sequences (**Figure** **4**a).

The hybrid coil was tested under two cases: 1) where the metamaterial dominates, resulting in a more concentrated SNR enhancement in the subcutaneous region, and 2) where the contributions from the metamaterial and coil are balanced, leading to improvements in both SNR and penetration depth. These images were compared with those obtained using the coil alone and the commercial single‐channel coil. The hybrid coil demonstrated superior performance, particularly in visualizing certain detailed features, such as the cartilage (white arrow).

Quantitative SNR comparisons were made across three distinct regions: two muscle segments and one bone segment. In the muscle region near the coil surface (red), case (1) provided an SNR enhancement of up to 2.43‐fold and 3.08‐fold compared to the coil alone and the commercial coil, respectively, while case (2) showed 1.85‐fold and 1.89‐fold improvements (Figure 4b). For the bone segment, which is located further away from the coil, both cases exhibited less pronounced SNR enhancement, with case (1) achieving up to 2.07‐fold and 2.27‐fold improvements, and case (2) showing 1.59‐fold and 1.72‐fold enhancements (Figure 4c). In the muscle region located ≈60 mm from the coil surface (blue), case (1) no longer offered an advantage over the coil alone, while case (2) still outperformed both alternatives with an SNR improvement of up to 1.29‐fold and 1.68‐fold (Figure 4d). These results highlight the hybrid coil's capacity to enhance SNR in both superficial and deeper tissue layers, offering versatility for various imaging applications.

## 4 Discussion

In this study, we introduce a strategy that integrates metamaterials with existing receive coils to create a hybrid coil, significantly enhancing the imaging capabilities of conventional coils. This approach achieves performance improvements that are otherwise unattainable with standard coils alone. The metamaterials are patterned on the same plane as part of the coil's conductor traces, thereby requiring no additional space while preserving the coil's original coverage. By meticulously manipulating the resonance frequencies of the metamaterial and coil individually, it is possible to detune both components from the MRI working frequency, resulting in a working mode of the combined hybrid structure that aligns with the MRI working frequency. In addition, both simulations and MRI validations demonstrate that precise control of the mutual coupling between the metamaterial and the coil enables the management of the trade‐off between the hybrid coil's peak SNR enhancement at the surface and overall penetration depth, allowing for the coil's improved imaging capabilities in various aspects.

When the metamaterial's frequency is tuned close to the Larmor frequency, it dominates the hybrid structure, resulting in a highly concentrated SNR in the close‐to‐surface region during phantom experiments, with an enhancement of up to 3.54‐fold compared to the coil alone within 40 mm above the coil. Traditionally, the most effective approach to achieve a higher maximum SNR level with a coil is by adopting smaller coil elements, and it often requires the construction of a coil array containing numerous channels to extend lateral coverage while maintaining high SNR. However, such array would require each coil element to be equipped with its own cabling, electronic panels, interfaces, adapters, and baluns, these components significantly add to the overall bulkiness and expense of the coil array. The proposed hybrid coil strategy allows for the creation of a wireless coil array, featuring multiple small wireless resonators coupled to a single receive loop. This approach substantially reduces the amount and cost of MRI RF components, making MRI hardware more accessible and affordable.

Moreover, by simply adjusting the tuning and matching elements, the hybrid coil can be reconfigured to balance the contributions from the coil and metamaterial, achieving an SNR enhancement of up to 2.35‐fold in phantom experiments, which persists to depths of 140 mm and beyond. This flexibility is advantageous for applications requiring an extended FOV. Furthermore, the hybrid coil's integration with metamaterials allows for easy adjustment of the metamaterial's resonance far away from the Larmor frequency, reverting the coil to its original state and functionality. We also demonstrate in ex vivo experiment that this hybrid coil provides substantial SNR improvements in the subcutaneous region, focusing on the fat or connective tissue beneath the skin, and can then be reconfigured to enhance SNR in deeper regions, improving the visualization of bone, cartilage, muscle, and other structures.

The proposed hybrid coil also exhibits limitations, particularly in achieving substantial SNR improvements at greater depths. This limitation stems from the compact dimensions of the metamaterial unit cells relative to the coil, which inherently restricts penetration depth. Consequently, for applications requiring deeper tissue visualization, such as spine or abdominal imaging with an extensive field of view, traditional coils may still offer superior performance. However, the hybrid coil's ability to adjust the metamaterial's resonance frequency further away from the Larmor frequency (as shown in Case (4)) allows it to effectively nullify any adverse influence from the metamaterial, ensuring no counterproductive impact on the coil's performance. In contrast, the hybrid coil holds particular promise for smaller, more localized imaging applications (e.g., brain, breast, or extremities), where proximity of the anatomy to the coil and the effects of multiple channels within a volumetric coil array collectively enhance SNR at the center of the ROI. Another potential challenge in implementing the hybrid coil in practical, real‐time MRI systems is maintaining precise tuning across both the coil and metamaterials. For the ease of adjustability, trimmer capacitors were employed to achieve the desired resonance states. For future designs, dedicated active tuning circuits, similar to those previously developed for programmable magnetic metamaterials,^[^ ^52^, ^53^ ^]^ could enable simultaneous, precise tuning across the coil and all metamaterial unit cells. Additionally, uniformity issues in the coil's sensitivity map caused by destructive interference or phase cancellation among metamaterial unit cells may be further addressed. Future work could explore a multi‐layer circuit design that introduces overlap between metamaterial unit cells, potentially minimizing these adverse effects and smoothing the coil's sensitivity map.

In conclusion, we present an alternative coil construction strategy that incorporates metamaterials into conventional loop receive coils, which can also be adapted to modify existing commercial coils for extended FOV and improved SNR. The current work demonstrates the feasibility and effectiveness of this construction technique in a single‐channel configuration, laying the groundwork for further advancements toward a multi‐channel configuration. In this setup, each channel will be paired with a metamaterial array to enable advanced imaging capabilities in targeted clinical trials. By combining parallel imaging techniques with such metamaterial‐enabled hybrid coil array design, additional improvements in imaging speed and resolution can be achieved. This approach holds the potential to facilitate the translation of advanced wireless metamaterial technologies into clinical MRI applications.

## 5 Experimental Section

### Coil Construction

The hybrid coil and reference coil were fabricated by milling 35‐µm thick copper cladding on a 1.5‐mm thick RF‐4 substrate using a printed circuit board prototype machine (ProtoMat S64, LPKF). The coil had an inner diameter of 150 mm and an outer diameter of 160 mm, with a copper trace width of 5 mm. Each unit cell in the 1 by 2 metamaterial array had inner and outer edge lengths of 36 and 40 mm, respectively, and a copper trace width of 2 mm.

### Numerical Simulation

All electromagnetic simulations were conducted using the frequency domain solver in CST Microwave Suite 2021. The dimensions used in the simulations for the hybrid coil and phantom matched those of the constructed coil and phantom.

### MRI Validation

For MRI validations, the BC served as the transmission coil, while the receiving coil was either the reference coil alone, the hybrid coil, or the Philips dStream Flex M coil. All phantom experiments were conducted using the gradient echo sequence, with an echo time (TE) of 4.6 ms and a repetition time (TR) of 100 ms. The FOV was 256 mm × 256 mm, the voxel size was 1 mm × 1 mm, and the slice thickness was 5 mm. For the ex vivo experiments, the FOV was 192 mm × 192 mm, the voxel size was 0.5 mm × 0.5 mm, and the slice thickness was 3 mm. TE and TR were 30 and 2500 ms for the PDw‐TSE scans, and were 10 and 450 ms for the T1w‐TSE scans.

## Conflict of Interest

The authors have filed patent applications on the work described herein, application No.: 16/002,458, 16/443,126, and 17/065,812. Applicant: Trustees of Boston University. Inventors: Xin Zhang, Stephan Anderson, Guangwu Duan, and Xiaoguang Zhao. Status: Active.

## Data Availability

The data that support the findings of this study are available from the corresponding author upon reasonable request.

## References

[1] P. C. Lauterbur , Nature 1973, 242, 190.

[2] N. K. Logothetis , Nature 2008, 453, 869. 18548064 10.1038/nature06976

[3] I. Young , Electron. Power 1984, 30, 205.

[4] M. H. Levitt , Spin Dynamics: Basics of Nuclear Magnetic Resonance, 2nd ed., John Wiley & Sons, Hoboken, NJ 2008.

[5] V. P. B. Grover , J. M. Tognarelli , M. M. E. Crossey , I. J. Cox , S. D. Taylor‐Robinson , M. J. W. McPhail , J. Clin. Exp. Hepatol. 2015, 5, 246. 26628842 10.1016/j.jceh.2015.08.001 PMC4632105

[6] U. Katscher , P. Börnert , C. Leussler , J. S. van den Brink , Magn. Resonance Med. 2003, 49, 144. 10.1002/mrm.10353 12509830

[7] P. Vernickel , P. Röschmann , C. Findeklee , K.‐M. Lüdeke , C.h. Leussler , J. Overweg , U. Katscher , I. Grässlin , K. Schünemann , Magn. Resonance Med. 2007, 58, 381. 10.1002/mrm.21294 17654592

[8] P. B. Roemer , W. A. Edelstein , C. E. Hayes , S. P. Souza , O. M. Mueller , Magn. Resonance Med. 1990, 16, 192. 10.1002/mrm.1910160203 2266841

[9] B. Gruber , M. Froeling , T. Leiner , D. W. J. Klomp , J. Magn. Resonance Imag. 2018, 48, 590. 10.1002/jmri.26187 PMC6175221 29897651

[10] C. J. Hardy , R. O. Giaquinto , J. E. Piel , K. W. Rohling AAS , L. Marinelli , D. J. Blezek , E. W. Fiveland , R. D. Darrow , T. K. F. Foo , J. Magn. Resonance Imag. 2008, 28, 1219. 10.1002/jmri.21463 18972330

[11] B. Keil , J. N. Blau , S. Biber , P. Hoecht , V. Tountcheva , K. Setsompop , C. Triantafyllou , L. L. Wald , Magn. Resonance Med. 2013, 70, 248. 10.1002/mrm.24427 PMC3538896 22851312

[12] J. P. Stockmann , T. Witzel , B. Keil , J. R. Polimeni , A. Mareyam , C. LaPierre , K. Setsompop , L. L. Wald , Magn. Resonance Med. 2016, 75, 441. 10.1002/mrm.25587 PMC4771493 25689977

[13] J. A. Nordmeyer‐Massner , N. D. Zanche , K. P. Pruessmann , Magn. Resonance Med. 2012, 67, 872. 10.1002/mrm.23240 22213018

[14] J. R. Corea , A. M. Flynn , B. Lechêne , G. Scott , G. D. Reed , P. J. Shin , M. Lustig , A. C. Arias , Nat. Commun. 2016, 7, 10839. 26961073 10.1038/ncomms10839 PMC5553354

[15] J. M. Vincent , J. V. Rispoli , IEEE Trans. Biomed. Eng. 2019, 67, 2187. 31794385 10.1109/TBME.2019.2956682 PMC7253317

[16] A. Port , R. Luechinger , L. Albisetti , M. Varga , J. Marjanovic , J. Reber , D. O. Brunner , K. P. Pruessmann , Sci. Rep. 2020, 10, 8844. 32483259 10.1038/s41598-020-65634-5 PMC7264329

[17] A. Port , R. Luechinger , D. O. Brunner , K. P. Pruessmann , Magn. Resonance Med. 2021, 85, 2882. 10.1002/mrm.28662 33433044

[18] E. Motovilova , T. Ching , J. Vincent , J. Shin , E. T. Tan , V. Taracila , F. Robb , M. Hashimoto , D. B. Sneag , S. A. Winkler , Sensors 2023, 23, 7588. 37688046 10.3390/s23177588 PMC10490642

[19] D. I. Hoult , Prog. Nucl. Magn. Reson. Spectrosc. 1978, 12, 41.

[20] G. Duan , X. Zhao , S. W. Anderson , X. Zhang , Commun. Phys. 2019, 2, 35. 31673637 10.1038/s42005-019-0135-7 PMC6822984

[21] X. Zhao , G. Duan , K. Wu , S. W. Anderson , X. Zhang , Adv. Mater. 2019, 31, 1905461. 10.1002/adma.201905461 PMC7108751 31663651

[22] A. V. Shchelokova , C. A. T. van den Berg , D. A. Dobrykh , S. B. Glybovski , M. A. Zubkov , E. A. Brui , D. S. Dmitriev , A. V. Kozachenko , A. Y. Efimtcev , A. V. Sokolov , V. A. Fokin , I. V. Melchakova , P. A. Belov , Magn. Resonance Med. 2018, 80, 1726. 10.1002/mrm.27140 29427296

[23] Z. Chi , Y. Yi , Y. Wang , M. Wu , L. Wang , X. Zhao , Y. Meng , Z. Zheng , Q. Zhao , J. Zhou , Adv. Mater. 2021, 33, 2102469. 10.1002/adma.202102469 34402556

[24] K. Wu , X. Zhao , T. G. Bifano , S. W. Anderson , X. Zhang , Adv. Mater. 2022, 34, 2109032. 10.1002/adma.202109032 PMC8831474 34865253

[25] K. Wu , X. Zhu , T. G. Bifano , S. W. Anderson , X. Zhang , Adv. Sci. 2024, 11, 2400261. 10.1002/advs.202400261 PMC11234395 38659228

[26] Y. Yi , Z. Chi , Y. Wang , M. Wu , L. Wang , D. Jiang , L. He , Y. Qi , X. Li , X. Zhao , Y. Meng , J. Zhou , Q. Zhao , Z. Zheng , Magn. Resonance Med. 2024, 91, 530. 10.1002/mrm.29870 37814581

[27] R. Schmidt , A. Slobozhanyuk , P. Belov , A. Webb , Sci. Rep. 2017, 7, 1678. 28490772 10.1038/s41598-017-01932-9 PMC5431866

[28] W. Lee , J. Lane , M. Febo , Y. K. Yoon , 2022 IEEE 72nd Electronic Components and Technology Conf. (ECTC), San Diego, CA, USA, June 2022.

[29] K. Wu , X. Zhu , X. Zhao , S. W. Anderson , X. Zhang , (Preprint) arxiv:2310.00153, v1, 2023.

[30] A. Shchelokova , V. Ivanov , A. Mikhailovskaya , E. Kretov , I. Sushkov , S. Serebryakova , E. Nenasheva , I. Melchakova , P. Belov , A. Slobozhanyuk , A. Andreychenko , Nat. Commun. 2020, 11, 3840. 32737293 10.1038/s41467-020-17598-3 PMC7395080

[31] X. Zhu , K. Wu , S. W. Anderson , X. Zhang , Adv. Mater. Technol. 2023, 8, 2301053.

[32] X. Zhu , K. Wu , S. W. Anderson , X. Zhang , Adv. Mater. 2024, 36, 2470244. 10.1002/adma.202313692 38569592

[33] K. Wu , X. Zhu , S. W. Anderson , X. Zhang , Sci. Adv. 2024, 10, eadn5195. 38865448 10.1126/sciadv.adn5195 PMC11168459

[34] M. Dubois , L. Leroi , Z.o Raolison , R. Abdeddaim , T. Antonakakis , J. de Rosny , A. Vignaud , P. Sabouroux , E. Georget , B. Larrat , G. Tayeb , N. Bonod , A. Amadon , F. Mauconduit , C. Poupon , D. Le Bihan , S. Enoch , Phys. Rev. X 2018, 8, 031083.

[35] A. Hurshkainen , A. Nikulin , E. Georget , B. Larrat , D. Berrahou , A. L. Neves , P. Sabouroux , S. Enoch , I. Melchakova , P. Belov , S. Glybovski , R. Abdeddaim , Sci. Rep. 2018, 8, 9190. 29907834 10.1038/s41598-018-27327-y PMC6003915

[36] V. Puchnin , V. Ivanov , M. Gulyaev , Y. Pirogov , M. Zubkov , IEEE Trans. Med. Imag. 2022, 41, 1587. 10.1109/TMI.2022.3143693 35030077

[37] M. Zubkov , A. A. Hurshkainen , E. A. Brui , S. B. Glybovski , M. V. Gulyaev , N. V. Anisimov , D. V. Volkov , Y. A. Pirogov , I. V. Melchakova , NMR Biomed. 2018, 31, e3952. 29944184 10.1002/nbm.3952

[38] M. Dubois , T. S. Vergara Gomez , C. Jouvaud , A. Ourir , J. de Rosny , F. Kober , R. Abdeddaim , S. Enoch , L. Ciobanu , J. Magn. Reson. 2019, 307, 106567. 31476633 10.1016/j.jmr.2019.106567

[39] I. Issa , K. L. Ford , M. Rao , J. Wild , IET Microw. Antenn. Propag. 2016, 10, 1378.

[40] I. Issa , K. L. Ford , M. Rao , J. M. Wild , IEEE Trans. Med. Imag. 2019, 39, 1085. 10.1109/TMI.2019.2942194 32054570

[41] D. I. Hoult , R. E. Richards , J. Magn. Reson. 1969, 24, 71.

[42] C. E. Hayes , L. Axel , Med. Phys. 1985, 12, 604. 4046995 10.1118/1.595682

[43] D. I. Hoult , Concepts Magn. Reson. 2000, 12, 173.

[44] W. Bakalski , W. Simburger , H. Knapp , H. D. Wohlmuth , A. L. Scholtz , 2002 IEEE MTT‐S Int. Microwave Symp. Digest, Vol. 1, IEEE, Piscataway, NJ 2002.

[45] Y. Zhu , C. R. Sappo , W. A. Grissom , J. C. Gore , X. Yan , IEEE Trans. Med. Imag. 2022, 41, 1420. 10.1109/TMI.2022.3140717 PMC9812758 34990352

[46] N. H. Fletcher , T. D. Rossing , The Physics of Musical Instruments, Springer Science & Business Media, Berlin 2012.

[47] A. P. Sample , D. T. Meyer , J. R. Smith , IEEE Trans. Industrial Electron. 2010, 58, 544.

[48] A. Kurs , A. Karalis , R. Moffatt , J. D. Joannopoulos , P. Fisher , M. Soljačić , Science 2007, 317, 83. 17556549 10.1126/science.1143254

[49] J. T. Vaughan , J. R. Griffiths , RF Coils for MRI, John Wiley & Sons, Hoboken, NJ 2012.

[50] P. Lorrain , D. R. Corson , Electromagnetic Fields and Waves, W. H. Freeman, San Francisco, 1970.

[51] R. W. Brown , Y. C. N. Cheng , E. M. Haacke , M. R. Thompson , R. Venkatesan , Magnetic Resonance Imaging: Physical Principles and Sequence Design, John Wiley & Sons, Hoboken, NJ 2014.

[52] T. S. Pham , H. N. Bui , J.‐W. Lee , J. Magn. Magn. Mater. 2019, 485, 126.

[53] H. N. Bui , T. S. Pham , J. S. Kim , J. W. Lee , J. Magn. Magn. Mater. 2020, 494, 165778.

